# Rectal adenocarcinoma resection specimen with an incidental carcinoid in the resection margin

**DOI:** 10.3892/ol.2014.1933

**Published:** 2014-03-04

**Authors:** JIE ZHOU, XIAODONG TENG

**Affiliations:** Department of Pathology, The First Affiliated Hospital, College of Medicine, Zhejiang University, Hangzhou, Zhejiang 310003, P.R. China

**Keywords:** carcinoid, small carcinoid, rectum, resection margin, second malignancy

## Abstract

Three cases of incidental carcinoid tumors have been identified in the surgical margin of rectal adenocarcinoma resection specimens. In all cases the rectal carcinoids exhibited low-risk features, such as a tumor size <10 mm, no muscularis propria invasion and no lymph node involvement. No further excision was conducted and the three cases were followed up for 38, 26 and 14 months, respectively. No regional or distant rectal carcinoid recurrence was identified. Occasionally this is inevitable in order to achieve a positive resection margin for the microcarcinoid during the surgical treatment of another malignancy. However, such carcinoids are usually low-risk and behave less aggressively than same-site adenocarcinomas. Thus, it appears reasonable to avoid further excision in patients who are undergoing a rectal adenocarcinoma resection that exhibits a positive margin for low-risk carcinoid tumor.

## Introduction

The term carcinoid is synonymous with the term well differentiated neuroendocrine tumor. Recently, carcinoids of the colon and the rectum have been grouped together. However, rectal carcinoids tend to behave less aggressively than colon carcinoids. Rectal carcinoids are often only diagnosed incidentally during routine lower endoscopy procedures due to the fact that such tumors are small, submucosal in location and rarely metastatize (1–3). In addition, rectal carcinoids are uncommon tumors. The age-standardized incidence of such carcinoids was ~0.3–1.2 cases per 100,000 individuals annually between 1992 and 1999, which has increased in recent decades due to improvements in diagnostic technology (such as endoscopy) and as a result of increased medical awareness (4). Rectal carcinoids tend to grow more slowly than same-site adenocarcinomas and harbor a favorable five-year survival rate. Gastrointestinal carcinoids are associated with a high incidence of second primary malignancy, found to occur in ~13.1% of all rectal carcinoids (4). The identification of an incidental gastrointestinal carcinoid during surgical treatment of another malignancy will usually only require resection without additional treatment, having little effect on prognosis (5). However, the treatment of unexpected carcinoids identified in the surgical margin of rectectomy specimens has not been previously described in any reported literature. In the present study three such cases have been presented and the current literature has been reviewed.

## Materials and methods

In total, three pathology reports, which revealed incidental carcinoids in the surgical margin of radical rectectomy specimens were retrieved from the pathology database of The First Affiliated Hospital (Hangzhou, China), from January, 2007 to April, 2013. Clinical details were acquired from medical records retrospectively, such details included patient gender, age, medical history, symptoms at presentation, modality of diagnosis and treatment. The pathological features were identified from the pathology reports and the carcinoid tumor size was reported as the largest diameter that was recorded microscopically. The follow-up was conducted via telephone interview and through examination of laboratory, endoscopy and imaging results that were obtained during clinical visits. The examination results were documented in the laboratory and imaging databases, which were also reviewed. This study was approved by the institutional ethics committee of The First Affiliated Hospital, College of Medicine, Zhejiang University (Hangzhou, China). Informed consent was obtained from the patients families.

## Results

### Patient and primary adenocarcinoma features

All three patients were male and were aged 62, 66 and 54 years at presentation. They all experienced hematochezia, leading to endoscopic evaluation and biopsy. This resulted in the identification of adenocarcinoma and subsequently a radical rectectomy (Dixon’s operation) was performed. Features of the primary adenocarcinomas are displayed in Table I.

### Incidental carcinoid features

**Figure 1:**
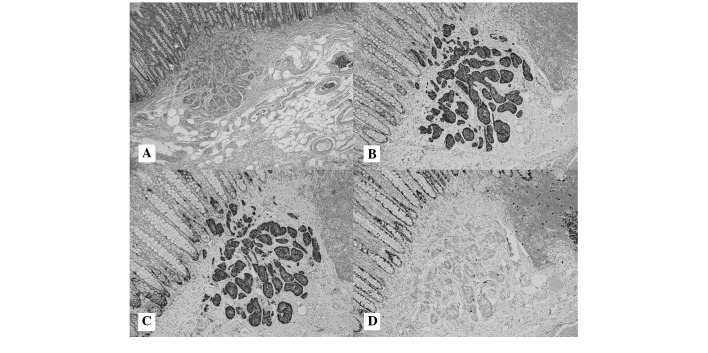
Immunohistochemical staining of carcinoid tumors. Stain, hematoxylin and eosin; magnification, ×50. (A) Small uniform tumor cells are arranged in small glands and have invaded the submucosal layer. Stain, envision two-step method; magnification, ×100. Tumor cells were markedly and diffusely positive for (B) synaptophysin and (C) chromogranin A, but negative for (D) Ki-67.

The distal margins of the resection specimens were 2–3 cm from the respective adenocarcinoma and were removed during surgical procedures. Sections of each distal margin revealed an unexpected microcarcinoid tumor within it. Their sizes were all <0.5 mm in diameter (0.2, 0.2 and 0.3 mm, respectively). All tumors invaded the submucosa only, without lymphovascular invasion, lymph node involvement or distant metastasis. Immunohistochemical staining was performed on all carcinoid tumors. The tumor cells were identified to be positive for synaptophysin, chromogranin A and cluster of differentiation 56, but were negative for Ki-67 (Fig. 1).

### Treatment and follow-up

Following diagnosis, patients were treated without further surgical or endoscopic resection. This decision was made by the patient and was based on the consideration that benign-like carcinoids have little effect on patient prognosis compared with adenocarcinoma. None of the patients underwent chemotherapy or radiation following the radical rectectomy. Patients were followed up for 38, 26 and 14 months, respectively. Case three suffered hepatic metastasis of the adenocarcinoma in the 13th month following the rectectomy and underwent partial resection of the liver. However, none of the three patients demonstrated carcinoid recurrence or metastasis.

## Discussion

In 2008, Landry *et al* (6) proposed a novel staging system for adenocarcinoma based on the assessment of 4,701 rectal carcinoid tumors, considering variables, such as tumor size, depth of invasion, lymph node involvement and distant metastasis. In the study, the five-year survival rates for patients with stage I, II, III or IV disease were 97, 84, 27 and 20%, respectively. Overall, patients with rectal carcinoids exhibit a favorable five-year survival rate (88.3%), as reflected by a large percentage of localized tumors (82%) (4). However, cancer-specific survival rates are comparable between rectal carcinoid and adenocarcinoma if tumors exhibit lymph node or distant metastasis (7). Furthermore, the metastatic rate of early-stage rectal carcinoids may be higher than those of rectal carcinomas if tumors are >10 mm in diameter (7,8).

Certain studies have identified various risk factors hypothesized to be involved in rectal carcinoid metastasis, such as a tumor size >10 mm, muscularis propria infiltration, the presence of lymphovascular invasion, the mitotic rate increased and the Ki-67 index increased (7, 9–12). Mani *et al* (3) evaluated >200 studies of rectal carcinoids and noted that tumor size and muscularis propria invasion were the two most important predictors of neoplastic malignancy. The majority of rectal carcinoids are <10 mm at the time of diagnosis with a median size of 6 mm (6). These lesions metastasized in <2% of patients (3). However, when a tumor reaches a size >10 mm, the metastatic rate increases notably. For example, in tumors measuring between 10 and 20 mm, the metastatic rate increases to 10–15%, whereas tumors measuring >20 mm have a metastatic rate of 60–80% (3). Another study showed that metastases were present in only 2% of tumors, which were <20 mm and did not exhibit muscularis propria invasion, compared with 48% in tumors that had invaded the muscularis layer (13). In addition, a large study in Asia revealed that a tumor size of >10 mm and the presence of lymphatic invasion were independently predictive of lymph node metastasis, whereas a tumor size >20 mm and the presence of venous invasion were independently predictive of distant metastasis (7).

Synchronous or metachronous secondary primary malignancies (SPM) are a common observation in carcinoid tumor patients (4,5,14–17). In addition, delayed metachronous SPM is common and incidence is eight times higher in carcinoid tumor patients compared with in the normal population (16). Of the gastrointestinal tract carcinoids, it was noted that a high percentage of associated tumors occurred with small intestinal carcinoids (29.0%), whereas a lower percentage was identified with rectal carcinoids (13.1%) (4). The majority of associated secondary malignancies were found to be located in the gastrointestinal tract (4,5,14–17), which occurred in 32–62% of tumors, followed by the genitourinary tract and the lung/bronchial system.

The etiology of this associated high risk of SPM remains unclear. It may be hypothesized that neuroendocrine factors secreted by carcinoids enhance the development or growth of other neoplastic tissue (18). In support of this hypothesis, higher levels of neuroendocrine factors are observed in small intestinal carcinoids (midgut) when compared with colorectal carcinoids (hindgut), in which SPMs are less common. However, it is difficult to explain the high incidence of delayed SPM following excision of carcinoid tumors. It is also speculated that carcinoid patients may exhibit an increased susceptibility to all forms of malignancy (16).

It is generally accepted that rectal carcinoids >20 mm in size require radical resection (19–22) due to the high rate of metastases (in ~3/4 cases) as described in the literature (13,14). However, controversy remains with regards to the management of tumors measuring <20 mm. For small rectal tumors (<10 mm), local removal is considered to be sufficient (20,22). However, certain tumors, including those measuring <5 mm, metastasize regionally and distally (7,8,23,24). In general, small rectal carcinoids are known as tumors with little metastatic risk, rendering local treatment desirable and reserving radical surgery for patients that display risk factors that are associated with metastasis. However, the aforementioned risk factors are difficult to identify during preoperative evaluation. In addition, the clinical role of preoperative imaging remains uncertain. Currently, treatment of small rectal carcinoids remains controversial as tumors measuring between 10 and 20 mm demonstrate unpredictable behavior and metastatic risk (20). However, it is reasonable to perform radical rectal resection for tumors, which are 10–20 mm in size and exhibit high-risk features, such as muscular and lymphovascular invasion (7,24).

The necessity for achieving a microscopically negative margin has been questioned in the past (25). Currently it is recommended that a negative margin is to be achieved if possible (24–26). Reasons for this are as follows: i) Malignancy criteria for rectal carcinoid are not well established and numerous benign-like tumors may evolve over a prolonged period of time resulting in late recurrences (24,27); ii) once a tumor has spread, chemotherapy and radiotherapy are not effective (19); whereas complete resection of the local disease offers the only chance of a cure; and iii) residual tumors may continue to release factors that enhance the development or growth of other neoplastic tissue.

The identification of an incidental gastrointestinal carcinoid during the operative treatment of another malignancy does not worsen the prognosis of the individual; complete resection is sufficient for therapy (5). However, occasionally it is difficult to identify minute rectal carcinoids during operative treatment of another malignancy, rendering a positive resection margin possible for the microcarcinoid. In the present study, no recurrence of carcinoids were observed in any of the three cases; however, during the follow-up period one patient suffered hepatic metastasis of adenocarcinoma in the 13th month following rectectomy. This indicated that it is reasonable to conduct a follow-up without further excision as such incidental microcarcinoids are usually absent of risk factors and are unlikely to recur prior to another malignancy.

Examination following resection of small rectal carcinoids is controversial. The latest consensus with regards to treatment guidelines for small rectal carcinoids is that tumors without lymph node involvement require no long-term follow up (21,22); however, exceptions do exist. Kwaan *et al* (24) reported that two patients with small rectal carcinoid tumors presented distant metastasis in the 5th and 13th year after resection. Conversely, small rectal carcinoids are usually treated by local excision at first, which renders it difficult to determine whether there is any lymph node involvement. In addition, synchronous or delayed SPM is a common finding in patients with carcinoid tumors. Thus, certain individuals may argue that long-term follow-up is recommended for delayed metastasis and secondary malignancies with a central point of focus on the gastrointestinal tract (17).

In conclusion, patients with rectal carcinoids, in particular those that are low-risk, have a favorable five-year survival rate. Such carcinoids behave less aggressively than same-site adenocarcinomas. Furthermore, the identification of an incidental rectal carcinoid during operative treatment of another malignancy is possible. However, it is possible to forgo further excision as incidental carcinoids are usually absent of risk factors and are unlikely to recur prior to another malignancy.

## Figures and Tables

**Table I:** Features of primary adenocarcinoma.

Case	Histological type	Differentiation	Tumor size (cm)	Depth of invasion	TNM stage
1	Adenocarcinoma	Intermediate	3.5×3.0	Within submucosa	T1N0M0
2	Adenocarcinoma	Intermediate	4.0×3.5	Within serosa^a^	T3N0M0
3	Adenocarcinoma	Intermediate	4.5×3.5	Within adventitia^b^	T3N0M0

a Tumor above peritoneal reflection.

b Tumor under peritoneal reflection.

TNM, tumor node metastasis.
